# Transplant Trial Watch

**DOI:** 10.3389/ti.2025.14820

**Published:** 2025-05-15

**Authors:** John O’Callaghan, John Fallon, Simon Knight

**Affiliations:** ^1^ Centre for Evidence in Transplantation, Nuffield Department of Surgical Sciences, University of Oxford, Oxford, United Kingdom; ^2^ University Hospitals Coventry and Warwickshire, Coventry, United Kingdom; ^3^ Oxford Transplant Centre, Churchill Hospital, Oxford, United Kingdom

**Keywords:** randomised controlled trial, liver transplantation (LT), kidney transplantation (KT), hypothermic oxygenated machine perfusion, immunosupression

To keep the transplantation community informed about recently published level 1 evidence in organ transplantation ESOT and the Centre for Evidence in Transplantation have developed the Transplant Trial Watch. The Transplant Trial Watch is a monthly overview of 10 new randomised controlled trials (RCTs) and systematic reviews. This page of Transplant International offers commentaries on methodological issues and clinical implications on two articles of particular interest from the CET Transplant Trial Watch monthly selection. For all high-quality evidence in solid organ transplantation, visit the Transplant Library: www.transplantlibrary.com.

**Randomised Controlled Trial 1**

Real-Time Biomarkers of Liver Graft Quality in Hypothermic Oxygenated Machine Perfusion.

*by Zhylko, A., et al. Journal of clinical medicine 2025; 14(2): 13.*

## Aims

To determine whether lactate concentration measured in real time during hypothermic oxygenated machine perfusion (HOPE) of liver grafts can serve as a biomarker to predict post-transplant graft function and early clinical outcomes.

## Interventions

Intervention Group (dHOPE): Liver grafts underwent dual hypothermic oxygenated machine perfusion for ≥120 min (portal vein + hepatic artery perfusion). Control (SCS): A separate arm of patients received conventional static cold storage. The paper’s focus is on 26 grafts in the dHOPE arm. During perfusion, perfusate was sampled every 30 min for lactate, oxygen, and flavin mononucleotide (FMN) measurements-up.

## Participants

Total Randomized Trial: 102 patients (26 allocated to dHOPE, 76 to standard cold storage), all receiving donor-after-brain-death (DBD) liver grafts. dHOPE Cohort Analyzed: 26 adult recipients meeting inclusion criteria (≥18 years old, informed consent). Median donor age was 53 years, and median recipient MELD was 12.

## Outcomes

Primary Outcome: Predictive value of perfusate lactate (and FMN) for Early Allograft Dysfunction (EAD) – a standard measure of post-transplant graft function. Secondary Outcomes: Correlation of perfusate biomarkers with post-transplant hospital and ICU stay, peak liver enzymes, post-transplant complications (e.g., Clavien-Dindo grade ≥3 events), and other composite clinical scores (MEAF, L-GrAFT7)

## Follow-Up

1 year posttransplantation.

## CET Conclusion

*by John Fallon*

The authors present analysis of a cohort of 26 patients within a single-centre RCT, the 26 patients received dHOPE and the control arm had standard care of SCS. The perfusate lactate during hypothermic preservation is utilised as a possible biomarker for transplant outcomes. Lactate assessment is feasible given it can be measured on a standard blood gas analyser, making it a quick and cheap biomarker compared with FMN, which requires a spectrofluorometer. Within the 26 patients they found lactate concentration in the perfusate after 120 min of dHOPE (≥3.45 mmol/L) predicted a significantly higher rate of EAD (67% vs. 6% below that threshold) and that elevated perfusate lactate correlated with longer hospital stays and higher peak transaminases, aligning with more severe graft dysfunction. Comparing lactate utility with FMN, which measured at 30 min also had predictive utility (AUC ∼0.83). It seems lactate best discriminated EAD after a longer perfusion (2 h), presumably reflecting time-dependent metabolic changes.

The study is limited by small sample size (n = 26 in dHOPE) and primarily DBD donors with relatively low-risk characteristics, which for a UK recipient cohort is less translatable. In the centres which use end-ischemic HOPE, it typically starts when the transplant is already in progress, so perfusate-based decisions to accept/reject might be limited. The absolute lactate level could be confounded by large graft weight, making delta-lactate (accumulation over 2 h) potentially more informative. Finally, methodologically there was no formal blinding described, but objective biomarker endpoints reduce detection bias.

Overall, while a secondary analysis of a relatively small single-centre randomised study, it was prospective with clearly defined endpoints and a structured HOPE protocol. Their findings reinforce that real-time lactate during HOPE could help gauge graft quality, complementing or substituting more complex measurements (e.g., FMN). This concept should be taken forward into a future multicentre validation, especially in broader donor populations (e.g., DCD, more steatotic grafts) and with extended HOPE. In a device to donor setting this may provide timing predictive information to influence clinical decision making.

## Trial Registration

ClinicalTrials.gov - NCT04812054.

## Funding Source

Non-industry funded.

**Randomised Controlled Trial 2**

Induction of Immune Tolerance in Living Related HLA-Matched Kidney Transplantation: A Phase 3 Randomized Clinical Trial.

*by Kaufman, D. B., et al. American Journal of Transplantation 2025 [record in progress].*

## Aims

This study aimed to investigate whether the MDR-101, a donor-derived cellular product, was able to induce immune tolerance compared to standard treatment in renal transplant patients.

## Interventions

Participants were randomised to either receive MDR-101 or standard immunosuppression.

## Participants

30 adult kidney transplant recipients from 2-haplotype human leukocyte antigen (HLA) –matched living siblings.

## Outcomes

The primary efficacy endpoint was the proportion of patients that achieved functional immune tolerance. Other outcomes measured were quality of life, adverse events, and renal and metabolic function.

## Follow-Up

36 months.

## CET Conclusion

*by Simon Knight*

This interesting multicentre randomised study investigated the ability of a donor stem-cell derived cell therapy product (MDR-101) to induce clinical tolerance in recipients of HLA-matched sibling renal transplants. Recipients received rATG induction and low-dose lymphoid irradiation post-transplant, followed by MDR-101 cell therapy on day 11. During the first-year post-transplant, immunosuppression was gradually withdrawn until tacrolimus was stopped completely at 1 year in patients with evidence of mixed chimerism. 75% of patients (15/20) remained IS-free for 2 years with an acceptable safety profile. Although small (just 30 patients), this is a challenging study to undertake, and the results are impressive. Application is currently limited to 2-haplotype matched siblings, but the ability to deliver therapy post-transplant provides scope to extend to previously transplanted eligible recipients.

## Jadad Score

3.

## Data Analysis

Modified intention-to-treat analysis.

## Allocation Concealment

Yes.

## Trial Registration

ClinicalTrials.gov - NCT03363945.

## Funding Source

Non-industry funded.

## Clinical Impact Summary

*by John O’Callaghan*

This is a well-written report of a very interesting clinical trial in renal transplantation. The results in terms of the successful withdrawal of immune suppression, and continued freedom from immune suppression, are very exciting.

The trial was conducted in a randomised, multicentre study, without blinding. The allogeneic cellular product “MDR-101” was tested in the induction of mixed chimerism and functional immune tolerance. Potential recipients were limited to those receiving their first transplant from a living related donor between 18 and 70 years. Donors were healthy adults with 2-haplotype HLA match with the recipient. Functional immune tolerance was defined as remaining off all immune suppressing drugs for at least 24 months, with no episodes of biopsy proven acute rejection, development of *de novo* DSA, GVHD, transplant loss of patient death. The sample size was small (20 patients in the study group and 10 patients in the control group) but this should not be taken as a criticism in this study.

A significant number of patients in the study group (19/20) established mixed chimerism for at least 6 months, this reduced to 56% by day 1095. Patients remained off immune suppression even if mixed chimerism was lost. At 24 months 15/20 patients in the study group were off immune suppression and 4/20 resumed immune suppression after complete withdrawal. The overall rates of adverse events were similar and there was no graft versus host disease.

The study significantly surpassed the FDA threshold for success, which was set at 48% functional tolerance for 2 years after withdrawal of immune suppression. There are limitations in terms of the selected patient population for this particular trial. However, with further testing and development, this study will mark a key point on the road towards transplantation without long-term immune suppression.

### Clinical Impact

4/5.

